# Ecology drives intragenomic conflict over menopause

**DOI:** 10.1111/ele.12208

**Published:** 2013-12-09

**Authors:** Francisco Úbeda, Hisashi Ohtsuki, Andy Gardner, Minus Baalen

**Affiliations:** 1 School of Biological Sciences, Royal Holloway University of LondonEgham, Surrey, TW20 0EX, UK; 2 Department of Evolutionary Studies of Biosystems, The Graduate University for Advanced StudiesShonan Village, Kanagawa, Hayama, 240-0193, Japan; 3 School of Biology, University of St AndrewsSt Andrews, KY16 9TH, UK

**Keywords:** Cancer, cooperation, demography, fertility, game theory, genomic imprinting, humans, hunter gatherers, kin selection, migration

## Abstract

Menopause is the transition from reproductive to non-reproductive life well before natural death. Rather than involving a smooth, rapid change, it is normally preceded by a long period of erratic hormonal fluctuation that is accompanied by a plethora of unpleasant symptoms. Here, we (1) suggest that this turbulent period owes to conflict, between a woman's maternally inherited (MI) and paternally inherited (PI) genes, over the trade-off between reproduction and communal care; (2) perform a theoretical analysis to show that this conflict is resolved either through silencing or fluctuating expression of one of the genes; (3) highlight which of the symptoms preceding menopause may result from antagonistic co-evolution of MI and PI genes; (4) argue that ecological differences between ancestral human populations may explain the variability in menopause among different ethnic groups; (5) discuss how these insights may be used to inform family planning and cancer risk assessment based on a woman's ancestral background.

## Introduction

Menopause is the end of female reproductive capability long before natural death (Prior 1998). A woman's fertility ends with her last menstruation at age ∼51, but her life extends to 65–70 years in societies where there is little access to modern medicine (Prior 1998; Jones *et al*. 2002). Menopause is extremely rare among mammals and has been confirmed beyond doubt only in humans and killer whales (Johnstone & Cant 2010). This phenomenon has attracted much attention from researchers in different fields who have suggested a number of explanatory hypotheses. Non-adaptationist hypotheses regard menopause as a side effect of ovarian senescence and extended human lifespan caused by modern medicine (Austad 1997), whereas adaptationist hypotheses suggest that it owes to natural selection (see Table 1). Some adaptationist hypotheses suggest that natural selection has favoured post-reproductive life because care provided by post-reproductive women increases the survival of kin and reproductive cessation reduces kin competition (Williams 1957; Alexander 1974; Hawkes *et al*. 1998; Cant & Johnstone 2008). In particular, the ‘Grandmother Hypothesis’ (Hawkes *et al*. 1998; Cant & Johnstone 2008; Johnstone & Cant 2010) posits that a young woman at the beginning of her reproductive career, being relatively unrelated to her neighbours, would maximise her inclusive fitness by focusing upon her own reproductive effort, whereas an older woman, being surrounded by her descendants, would maximise her inclusive fitness by giving up her own reproduction and providing care for others. This hypothesis has gained empirical support in recent years (Lahdenpera *et al*. 2004, 2012).

**Table 1:** Theories of menopause

Explanation	Summary	References
Extended Lifespan	Recent extension of human lifespan results in women living beyond reproductive age	(Austad 1997)
Good Mother	Trade-off between giving up own reproduction (when there is a growing age-related mortality) in exchange of greater survival of depending children	(Williams 1957; Alexander 1974)
Grandmother Hypothesis	Menopause is the result of a trade-off between giving up own reproduction in exchange of:	
	(1) Greater survival of kin	(Hawkes *et al*. 1998)
	(2) Reduced mate competition with relatives	(Cant & Johnstone 2008; Johnstone & Cant 2010)
Better Longer	Post-reproductive lifespan results from unidirectional selection against death before completion of reproductive life	(Tully & Lambert 2011)

Summary of evolutionary explanations for menopause.

**Figure 1:**
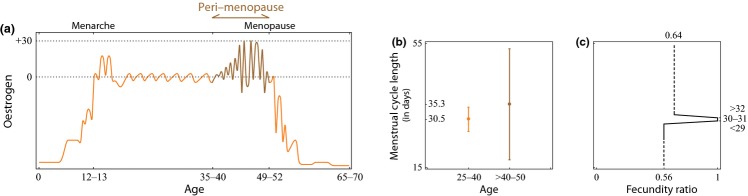
Menopause and peri-menopause endocrinology. (a) Schematic representation of oestrogen levels during a woman's lifetime [adapted from Prior (2006) and Douma *et al*. (2005)]. Menarche occurs between ages 12–13 (Butts & Seifer 2010), peri-menopause begins between ages 35 and 40 (Douma *et al*. 2005; Voorhuis *et al*. 2010) and terminates with menopause at age 51 (Butts & Seifer 2010), the life expectancy is 65–70 among modern hunter gatherers with no access to modern medicine (Jones *et al*. 2002). (b) Length of menstrual cycles at different ages. The average age and standard errors are provided. (Ferrell *et al*. 2005). (c) Fertility as a function of cycle length (Small *et al*. 2006).

However, it is not clear why an adaptive menopause should be such a long and difficult process, rather than a rapid and smooth transition from the reproductive to the non-reproductive phases of a woman's life. Specifically, menopause is preceded by a peri-menopausal period of 10–15 years, during which fertility decline is not driven by the demise of hormone production due to ovarian senescence but rather by dramatic fluctuations in oestrogen levels, resulting in irregular menstrual cycles and skipped ovulations (Prior 1998, 2006; Douma *et al*. 2005) (Fig. 1). Indeed, oestrogen reaches its highest levels in a woman's lifetime during peri-menopause; as high as 30% above the average level during the regular reproductive period (Santoro *et al*. 1996; Prior 1998, 2006; Douma *et al*. 2005) (Fig. 1). This hormonal deregulation during peri-menopause results in a plethora of unpleasant symptoms, including psychological (e.g. mood-swings, depression), vaso-motor (e.g. hot flashes, night sweats) and reproductive (e.g. painful intercourse, vaginal dryness) disorders (Gold *et al*. 2000; Avis *et al*. 2001). Interestingly, these symptoms often appear to be working at cross-purposes (Prior 1998, 2006). Duration, hormonal chaos and contradictory symptoms, taken together, suggest that peri-menopause is a detrimental process, from a woman's perspective.

Moreover, the turbulent physiology of peri-menopause and the timing of menopause do not appear to owe to constraint, as both phenomena are highly variable among different ethnic groups (Avis *et al*. 2001; Gold *et al*. 2001; Butts & Seifer 2010). For example, even after controlling for confounding variables: African Americans experience a longer peri-menopause, with intensified vaso-motor symptoms, that terminates in earlier menopause, whereas Japanese Americans experience a shorter peri-menopause, with milder vaso-motor symptoms, that terminates in later menopause (Gold *et al*. 2000, 2001; Avis *et al*. 2001; Henderson *et al*. 2008; Butts & Seifer 2010). In particular, vaso-motor symptoms are 2.5 times more prevalent among African Americans (1.56%) than among Japanese Americans (0.63%) (Gold *et al*. 2001). Furthermore, premature ovarian failure (i.e. menopause before the age of 40) is 10 times more prevalent among African Americans (1.4%) than among Japanese Americans (0.14%) (Luborsky *et al*. 2003).

Here, we suggest that the duration and turbulent nature of peri-menopause owes to intragenomic conflict between a woman's maternally inherited (MI) and paternally inherited (PI) genes. Intragenomic conflict refers to instances in which natural selection favours genes with opposing phenotypic effects expressed in the same individual, and has been associated with several medical disorders (Wilkins & Úbeda 2011). We show that the ecological factors which, according to the Grandmother Hypothesis (Hawkes *et al*. 1998; Cant & Johnstone 2008), have driven the evolution of a menopause in humans, may also drive intragenomic conflicts over reproductive schedules. In particular, as a result of female-biased dispersal and/or male-biased adult mortality or variance in reproductive success, a woman's PI genes are favoured to promote an earlier menopause, to the benefit of her social group, while her MI genes are favoured to promote a later menopause, to the detriment of her social group. Under this view, the period of active intragenomic conflict defines the turbulent peri-menopausal stage of a woman's life.

We develop a ‘battleground model’ to assess how this conflict between a woman's MI and PI genes unfolds as she ages. We also develop two ‘resolution models’, founded upon the biology of fertility, to assess possible outcomes of this conflict. We use these models to make explicit predictions about the imprinting status of genes that underpin menopause in general and primary ovarian insufficiency in particular. We also show that the erratic hormonal fluctuations (and related symptoms) experienced by peri-menopausal women are a suggestive of such conflict. These models allow us to link, for the first time, the symptoms of menopause to ancestral human ecology. We discuss the implications for personalised family planning and medicine.

## Materials and Methods

We employ an inclusive-fitness model to explore selection acting on genes with different parental origin coding for fertility in females. Models that explore the existence of conflict between evolutionary agents are termed battleground models. Having established the conditions under which genes with different parental origin might be in conflict, we employ two game theory models to explore the evolutionary outcomes of this conflict. These models differ in the mechanisms for controlling fertility. Models that explore the outcome of conflict between evolutionary agents are termed resolution models.

### Battleground model

We build upon the model of Úbeda & Gardner (2012). This considers an infinite population of diploid individuals subdivided into social groups. Upon reaching maturity, women and men disperse to new groups with probabilities *d*_*f*_ and *d*_*m*_ respectively, and otherwise remain in their natal groups. This allows consideration of any type of residential system, including matrilocality (*d*_*f*_ < *d*_*m*_), bilocality (*d*_*f*_* *= *d*_*m*_) and patrilocality (*d*_*f*_* *>* d*_*m*_). Mating follows dispersal, and occurs at random between women and men within each group. The probabilities that two random juveniles born in the same group share the same mother and same father are α and β, respectively. This allows consideration of any type of reproductive system, including monogamy (α ≈ β), polygyny (α < β) and polyandry (α > β) (Úbeda & Gardner 2012).

Whereas Úbeda & Gardner (2012) considered generic social traits, here we focus on reproductive traits. We consider that a woman can allocate her limited resources either into raising juveniles in her social group or else into producing her own offspring. By increasing her investment into raising neighbouring juveniles, a woman improves their survival to adulthood by an amount *b* while reducing the number of her own children she would otherwise have raised to adulthood by an amount *c*. We are interested in finding out how a woman's valuation of neighbouring juveniles relative to the production of her own offspring changes as she ages. This is a combination of the coefficient of relatedness of a woman of age *t* to her neighbouring juveniles *r*_*Ax*|*t*_ , and the level of competition *a* between related individuals. First, we determine the condition for selection to favour a reduction in fertility of a woman of age *t*, and write this as *c*/*b *< *S*_*t*_ , where *S*_*t*_ defines the ‘potential for sterility at age *t* ’. That is, menopause is selectively favoured when *S*_*t*_ is above the threshold 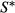
= *c*/*b*, which we term the ‘menopause threshold’.

More specifically, we separate the potentials for sterility at age *t* when the gene expressed is MI 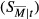 vs. PI 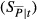:


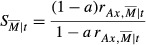
(1a)


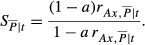
(1b)

**Figure 2:**
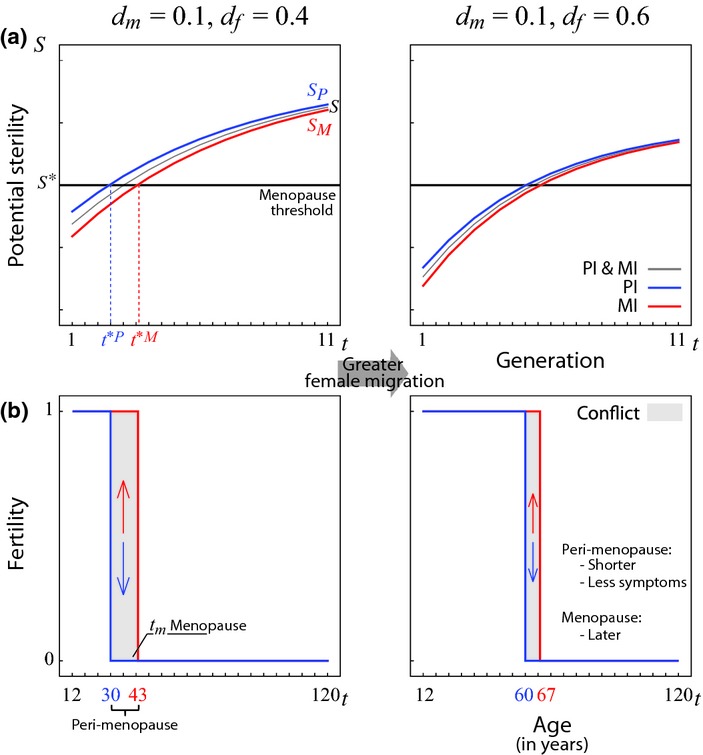
Conflict over menopause. (a) Potential for sterility of the MI gene (red), PI gene (blue), and MI and PI combined (black) as a function of age. (b) Intragenomic conflict as a function of age. Before the age t^*P^ natural selection favours genes that promote fertility both when MI and PI (no conflict). Between the ages t^*P^ and t^*M^ natural selection favours genes that promote fertility when MI but sterility when PI (conflict). From t^*M^ on, natural selection favours genes that promote sterility both when MI and PI (no conflict). To elaborate (a and b) we assume that α = β = 0.1 and μ_*m*_ = μ_*f*_ = 0.1 and we consider two cases of female-biased dispersal: less female bias when *d*_*m*_ = 0.1 and *d*_*f*_ = 0.4, and more female bias when *d*_*m*_ = 0.1 and *d*_*f*_ = 0.6.

These expressions correspond to eqns (A.01–A.14) of Úbeda & Gardner (2012). Substituting 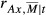, 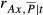 and *a* into 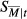 and 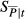 — our eqns (1a) and (1b) respectively — yields recursions for the potentials for sterility via the MI and PI genes in terms of the ecological parameters: dispersal (*d*_*m*_, *d*_*f*_ ), mating system (α, β) and mortality (μ_*m*_*,* μ_*f*_ ). Iterating these recursions, we calculate the potentials for sterility via the MI and PI genes as functions of the age of the focal female, given a set of numerical values for the ecological parameters (method used to generate Fig. 2).

### Resolution models

The battleground model establishes the ecological conditions under which there is a conflict of interest between a woman's MI and PI genes. Here, we develop two game theory models to investigate the antagonistic interaction between MI and PI genes. The difference between these two models lies in the different modes by which a woman's genes control her fertility.

#### Directional control

For the purpose of biological concreteness and illustration, we consider the role played by the number of primordial ovarian follicles on a woman's fertility. Ovaries carry a stock of ∼300 000 ovarian follicles by 5 months of gestational age, which then undergoes a decline throughout a woman's life (Wallace & Kelsey 2010). Menopause is caused by the depletion of the ovarian follicle stock, and occurs at age ∼51 when ∼1000 follicles remain (Wallace & Kelsey 2010). However, this decline is not uniform: at age ∼38, when ∼16 000 ovarian follicles remain, there is a marked increase in the rate of ovarian follicle attrition (Richardson *et al*. 1987; Faddy *et al*. 1992; Wallace & Kelsey 2010), a phenomenon that is unknown in other mammals (Jones & Krohn 1961; Gosden & Telfer 1987; Nichols *et al*. 2005; Jones *et al*. 2007).

We consider a locus that modifies a woman's fertility by altering the stock or rate of decline of ovarian follicles (atresia). We denote the expression levels of the MI and PI genes by 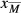 and 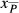 respectively. We assume that these combine additively to determine the total level of expression of that locus, i.e. 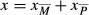.

We consider that fertility is a function of the total level of expression of this gene, i.e. *f *= *F*(*x*). We use the term ‘fertility inhibitor’ to refer to any locus whose greater expression reduces the stock (or accelerates atresia) of ovarian follicles, inhibiting fertility (Fig. A2). In contrast, we use the term ‘fertility enhancer’ to refer to any locus whose greater expression augments the stock (or slows atresia) of ovarian follicle, enhancing fertility (Monget *et al*. 2012) (Fig. A2). For simplicity, we model a fertility enhancer but symmetrical results can be derived for a fertility inhibitor.

We assume that the payoffs 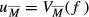 and 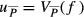 — gained by the MI and PI genes respectively — are unimodal functions, with 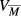 and 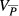 reaching their maxima at 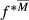 and 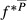 respectively (Fig. A2-i). For simplicity, we assume that the MI gene is favoured for greater fertility than the PI gene, i.e. 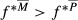 (Fig. A2; symmetrical results can be derived when the direction of the conflict is reversed, i.e. 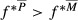.

Consequently, the payoffs for the MI and PI genes are functions of the total level of expression, i.e. 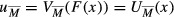 and 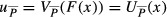. Let 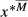 and 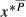 be the total levels of expression that maximise fertility for the MI and PI copies, respectively, i.e. 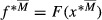 and 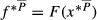 (Fig. A2). From our assumption that 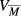 and 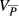 are unimodal it follows that 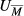 and 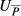 are also unimodal, with 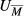 and 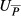 reaching their maxima at 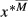 and 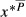 respectively. From our assumptions that the locus is a fertility inhibitor and the sense of conflict being 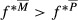 it follows that 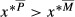 (Fig. A2).

In particular, we use Gaussian payoff functions, which satisfy the above assumptions:


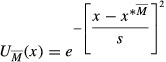
(2a)


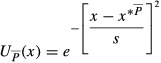
(2b)

where *s* determines the variance of both functions.

#### Stabilising/destabilising control

For the purpose of biological concreteness and illustration, we consider the role played by hormonal levels and menstrual cycle length on a woman's fertility. Four hormones are mainly responsible for a woman's menstrual cycle, namely oestrogen and progesterone, produced by the ovaries; and luteinising hormone (LH) and follicle-stimulating hormone (FSH), produced by the pituitary gland (Prior 1998). The levels of these hormones alternate regularly during a woman's reproductive life (Prior 1998), and their relative levels determine the duration of the menstrual cycle (Landgren *et al*. 1980). In particular, higher levels of oestrogen result in relatively short cycles, whereas lower levels of oestrogen result in relatively long cycles (Landgren *et al*. 1980).

The regularity in the alternation of these hormones results in minimal cycle length variability during a woman's reproductive life (ages 15–40) with an average duration of ∼30 days per menstrual cycle (Vitzthum 2009) (Fig. 1). Upon entering peri-menopause, levels of oestrogen increase and begin to fluctuate erratically (Prior 1998). Oestrogen disrupts the regular alternation of hormones, and menstrual cycles shorter or longer than 30 days become increasingly frequent (Prior 1998). Fertility is maximised when menstrual cycles are regular (i.e. every 30 days) and declines if menstrual cycles become irregular [i.e. before or after 30 days (Small *et al*. 2006); Fig. 1].

We consider a locus that modifies a woman's fertility by altering the rate of production of hormones during the menstrual cycle and thus altering its length. We denote the levels of expression of the MI and PI genes by 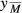 and 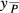 respectively. We assume that these combine additively to determine the total level of expression of that locus, i.e. 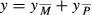.

We assume that fertility is a function of the total level of expression of this gene, i.e. *f* = *F*(*y*). We use the term ‘fertility maximiser’ to refer to any locus whose default expression maximises fertility. That is, its enhanced expression reduces the length of the menstrual cycle, reducing fertility; and its inhibited expression increases the length of the menstrual cycle, also reducing fertility (Fig. A2). The fertility function *F* is thus unimodal, reaching a single maximum at 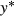. Notice that this, in practice, corresponds to any locus that mediates the length of a woman's menstrual cycle (Fig. 1). For completeness, we use the term ‘fertility minimiser’ refer to any locus whose regular expression minimises fertility. That is, both its enhanced and its inhibited expression reduce fertility (Fig. A2). For simplicity, we model a fertility maximiser but symmetrical results can be derived for a fertility minimiser.

We assume that the payoffs 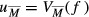 and 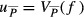 — gained by the MI and PI genes respectively — are unimodal with 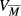 and 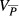 reaching their maxima at 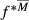 and 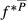 respectively (Fig. A2-ii). For simplicity, we assume that the MI gene is favoured for greater fertility than the PI gene, i.e. 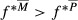 (Fig. A2-ii; symmetrical results can be derived when the direction of the conflict is reversed, i.e. 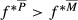.

Consequently, the payoffs functions for the MI and PI genes are functions of the total level of expression, i.e. 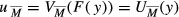 and 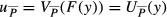 respectively. Let 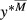 and 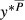 be the total levels of expression that maximise fertility for the MI and PI copies, respectively, i.e. 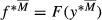 and 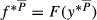. We assume that the total level of expression that maximises fertility for the PI copy corresponds to the fertility maximum, i.e. 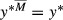 (Fig. A2-ii-a). From the assumptions that the locus is a fertility maximiser and 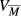 and 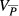 are unimodal, it follows that 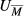 is unimodal with its maximum at 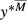 but 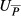 is bimodal, with two maxima at 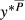 and 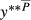. From the assumptions that the locus is a fertility maximiser and 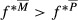, it follows that 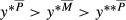 (Fig. A2).

In particular, we use the following Gaussian functions that satisfy the above assumptions:


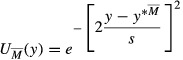
(3a)


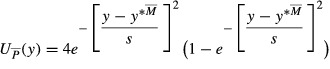
(3b)

#### Adaptive dynamics

We assume that level of gene expression at any time is a random variable with a uniform probability distribution over a range of values defined by their lower and upper bounds. In particular, the window of expression is 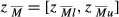 for the MI gene and 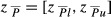 for the PI gene, where *z* ∈ [*x*,*y*]. When lower and upper bounds coincide (*z*_*l*_* *≈ *z*_*u*_), the genes exhibit the same level of expression in each menstrual cycle. When lower and upper bounds differ (*z*_*l*_ ≠ *z*_*u*_), the gene may exhibit a different level of expression from one menstrual cycle to the next.

Because the levels of expression of the MI and PI genes may fluctuate stochastically, the payoff of the MI and PI genes are given by the expectation taken over all possible pairs of levels of expression, that is,


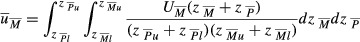
(4a)


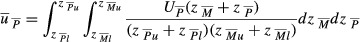
(4b)

The adaptive dynamics of the window of expression of a gene is given by the dynamic system:


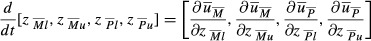
(5)

**Figure 3:**
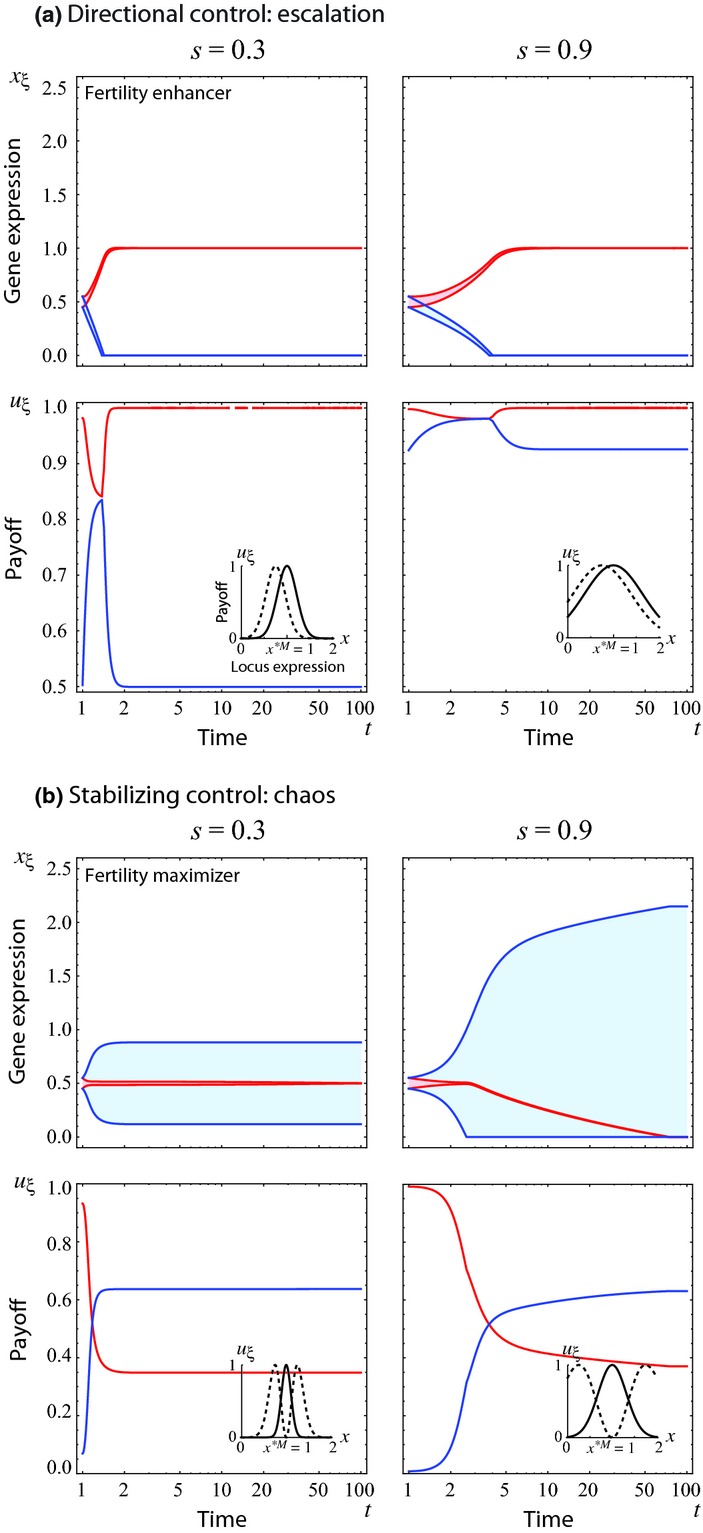
Adaptive dynamics of gene expression. We consider the cases when gene expression exerts: (a) directional control of fertility (in particular, a fertility inhibitor), with payoff functions characterised by x^*M^ = 1.00, x^*P^ = 0.75 and *s *=* *0.3 or *s *=* *0.9; and (b) stabilising control of fertility (fertility maximiser), with payoff functions characterised by x^*M^ = 1.00 and *s *=* *0.3 or *s *=* *0.9. In each case, the first row represents the evolution of parent-of-origin-conditional gene expression over time. We consider that gene expression can take any value within a range (shadowed area) determined by its lower and upper bounds (continuous lines) with equal probability (uniform probability density). We assume that there are initially no differences between the MI and PI genes, and both are expressed within the range [0.45, 0.55] whose combined expression [0.90, 1.10] is approximately optimal for the MI gene, x^*M^ = 1. The second row represents the evolution of parent-of-origin expected payoff over time. Insets illustrate the payoff functions.

We assume that there is a lower bound at 0 in our evolutionary dynamics, since gene-expression levels cannot be negative. In practice, this is done by fixing negative time-derivatives to 0 as required. Given a set of initial conditions, we use equation (5) to generate Fig. 3.

Qualitatively similar results are obtained using an alternative modelling approach, namely formulating payoff matrices for each gene and finding the evolutionarily stable strategy (ESS) for each game (see Appendix).

## Results

### Battleground model

For consistency with the Grandmother Hypothesis, and for the purpose of concreteness and illustration, we will focus on female-biased dispersal as the driver of intragenomic conflict over menopause. However, the same results will be found for any ecology resulting in females being more related to her neighbours through their PI genes (namely, male-biased adult mortality and/or variance in reproductive success), and symmetrical results for any ecology resulting in females being more related to their neighbours through their MI genes (namely, female-biased adult mortality and/or variance in reproductive success).

Consistent with the Grandmother Hypothesis (Cant & Johnstone 2008), we find that female-biased dispersal (*d*_*f*_ > *d*_*m*_) with monogamy (α ≈ β) leads to women having a lower potential for sterility early in reproductive life, which then monotonically increases with age (i.e. *S*_*t*+1_ > *S*_*t*_ for all *t* ; Fig. 2). Moreover, this qualitative pattern also holds both for MI and PI genes (i.e. 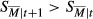 and 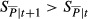 for all *t*; Fig. 2). However, the potential for sterility is always higher for a woman's PI genes and lower for her MI genes (i.e. 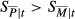 for all *t*; Fig. 2). Consequently, the menopause threshold will be crossed at different ages for these two sets of genes (i.e. 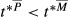 such that 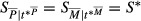, resulting in intragenomic conflict over fertility during the intervening ages (Fig. 2). That is, for 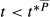, both sets of genes are favoured to maintain fertility (no conflict); for 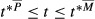, the PI genes are favoured to reduce fertility while MI genes are favoured to increase fertility (conflict); and for 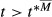, both sets of genes are favoured to suppress fertility (no conflict; Fig. 2).

Our model predicts that there is conflict between PI and MI genes during the approach to menopause (peri-menopause) (Fig. 2). We suggest that peri-menopause initiates when the PI genes cross their menopause threshold (i.e. at age 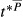) and terminates in actual menopause by the time that the MI genes cross their menopause threshold (i.e. at age 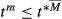). Hence, intragenomic conflict could play an important role in explaining the unpleasant symptoms observed during peri-menopause.

Finally, our model predicts that greater female-biased dispersal (*d*_*f*_′ > *d*_*f*_ > *d*_*m*_) leads to a shorter window of conflict preceding menopause 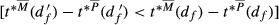, and a smaller degree of conflict 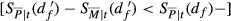 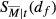 for 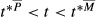; Fig. 2]. We therefore predict that, in populations with greater female-biased dispersal, women will experience shorter and less symptomatic peri-menopause. Our model also predicts that in populations with greater female-biased dispersal women experience menopause later in life (Fig. 2).

### Resolution models

#### Directional control and escalation

**Figure 4:**
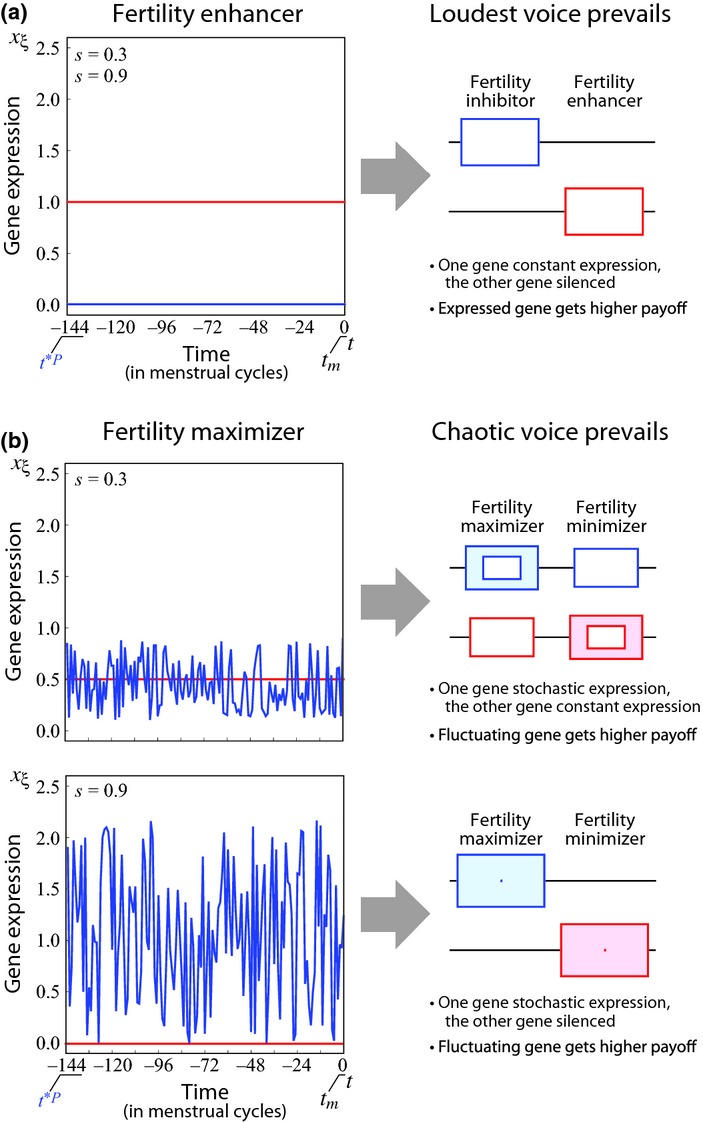
Resolution of conflict over menopause. We consider the cases when: (a) Gene expression exerts directional control of fertility (in particular a fertility inhibitor); (b) Gene expression exerts stabilising control of fertility (fertility maximiser). We illustrate the cases when *s *=* *0.3 and *s *=* *0.9 respectively. In each case, the first column presents the ESS level of expression for the MI (red) and PI (blue) genes during peri-menopause. The second column presents the schematic representation of the ESS gene expression for each copy and each type of gene.

When the locus is a fertility enhancer, our battleground model predicts that natural selection will favour a greater level of expression when the gene is MI (e.g. increasing fertility by preserving ovarian follicles) and a lower level of expression when the gene is PI (e.g. decreasing fertility by destroying ovarian follicles). Our resolution model shows that every increase in the level of expression of the MI gene is followed by a decrease in the level of expression of the PI gene (Fig. 3a). The end-point of this escalation is the self-imposed silencing of the PI gene and the expression of the MI gene at its optimal level (Fig. 4a). In contrast, when the locus is a fertility inhibitor, our battleground model predicts that natural selection will favour a greater level of expression when the gene is PI but a lower level of expression when the gene is MI. The outcome of this conflict is self-imposed silencing of the MI gene and expression of the PI gene at its optimal level. These results recover the ‘loudest voice prevails’ principle (Haig 1996).

Thus, our models predict that genes mediating fertility in a directional mode (i.e. fertility enhancers and fertility inhibitors) are likely to exhibit parent-of-origin differential expression with silencing of one of the genes (classic genomic imprinting). Moreover, our model predicts the direction of this asymmetry in expression: paternally silenced and maternally expressed if the gene product increases fertility during peri-menopause and delays menopause (e.g. via decelerated follicle destruction), and the reverse if the gene product reduces fertility during peri-menopause and hastens menopause (e.g. via accelerated follicle destruction) (Fig. 4a). While our model provides a resolution for the intralocus conflict, interlocus conflict between maternally expressed fertility enhancers and paternally expressed fertility inhibitors persists.

#### Stabilising/destabilising control and chaos

When the locus is a fertility maximiser (in the case of genes controlling the length of menstrual cycles via oestrogen production; Fig. 1), our battleground model predicts that natural selection will favour changing the level of expression when the gene is PI (e.g. decreasing fertility by changing the length of the menstrual cycle) but maintaining the level of expression when the gene is MI (for example, maintaining fertility by maintaining the length of the menstrual cycle). Our resolution model shows that every increase in the stochasticity of expression of the PI gene is countered by a decrease in the stochasticity of expression of the MI gene (Fig. 3b). The end-point of this co-evolutionary process is stochastic expression of the PI gene within the range of values that attains the PI gene's desired fertility, and constant expression of the MI gene at a level equal to or lower than its original (Fig. 4b). Whether MI genes are ultimately silenced depends on whether the lower bound of the range of values that maximise the PI gene's fertility is sufficiently low (Fig. 4b *s *=* *0.3) or not (Fig. 4b *s *=* *0.9).

In contrast, when the locus is a fertility minimiser, natural selection will favour changing the level of expression when the gene is MI but maintaining the level of expression when the gene is PI. The outcome of this conflict is a constant expression of the PI gene at equal or lower level than its original one, and the stochastic expression of the MI gene within the range of values that attains the MI gene's desired fertility. Whether PI genes are ultimately silenced depends on whether the lower bound of the range of values that maximise the MI gene's fertility is positive. As an analogy to the loudest-voice-prevails principle, we term this the ‘chaotic-voice-prevails’ principle.

Thus, our models predict that genes mediating fertility in a stabilising/destabilising mode (i.e. fertility maximisers and fertility minimisers) are likely to exhibit parent-of-origin differential expression with stochastic expression of one copy (a novel form of genomic imprinting). In particular, our model predicts the direction of this asymmetry: constant expression when MI but stochastic expression when PI, if changes in the gene product decrease fertility during peri-menopause (e.g. via hormonal fluctuations that shorten or lengthen the interval between menstrual cycles); and constant expression when PI but stochastic expression when MI, if changes in the gene product promote fertility during peri-menopause (Fig. 4b).

## Discussion

We have shown that the ancestral ecology responsible for driving the evolution of human menopause, according to the Grandmother Hypothesis, may also drive intragenomic conflict between a woman's MI and PI genes over her fertility during peri-menopause and the timing of her menopause. We suggest that a woman's menopause is characterised by the struggle between her PI genes, which are favoured to end her reproductive capacity, and her MI genes, which are favoured to maintain her reproductive capacity. Under this view, several of the unpleasant symptoms associated with peri-menopause may be explained by intragenomic conflict arising as a consequence of human ecology.

As a result of this intragenomic conflict, we predict that genes underpinning peri-menopause will show different patterns of expression when MI and PI. Specifically, we predict that genes mediating fertility during the menopausal transition will exhibit two patterns of expression: (1) silencing of one copy and expression of the other, when determining fertility in a directional manner (resulting from the loudest-voice-prevails principle); and (2) stable expression of one copy and random expression of the other, when determining fertility in a stabilising manner (resulting from the chaotic-voice-prevails principle).

If ancestral human populations exhibited female-biased dispersal, then we predict that those genes whose greater expression promotes fertility (fertility enhancers) during peri-menopause will be paternally silenced and maternally expressed, whereas those genes whose greater expression reduces fertility (fertility inhibitors) will be maternally silenced and paternally expressed. This might be the case for genes mediating the stock of ovarian follicles and the rate of atresia at the onset of peri-menopause, when follicle destruction accelerates (Cant & Johnstone 2008). These predictions provide a first evolutionary explanation for why autosomal genes *GNAS1*, *DLX5* and *WT1-AS* controlling ovarian follicle stock as fertility enhancers appear to be maternally expressed and paternally silenced in agreement with our model predictions (see Table 2 and references therein). Recently, the maternally expressed gene *KCNQ1* (Wilkins & Úbeda 2011) has been associated with age of menopause (Spencer *et al*. 2013), although its specific role remains unknown. In addition, conflict between fertility-enhancing and fertility-inhibiting loci can explain the overexpression of antagonistic genes that mediate fertility. This could provide an explanation for those menopausal symptoms that appear to work at cross-purposes.

**Table 2:** Imprinted genes linked to fertility and menopause

Gene	Imprint status-Fertility-Imprint prediction	References
*GNAS1*	Direct evidence shows that gene *GNAS1* is imprinted in some human tissues, in particular maternally expressed and paternally silenced in the pituitary gland and ovaries.	(Hayward *et al*. 1998; Mantovani *et al*. 2002; Wilkins & Úbeda 2011)
	A loss-of-function mutation of *GNAS1* results in follicle-stimulating hormone (FSH) resistance (among other symptoms) when the gene is MI but not when it is PI. FSH resistance results in reduced ovarian follicle stock and premature ovarian failure. Thus a loss-of-function mutation of *GNAS1* results in premature ovarian failure when MI but not when PI. Greater expression of *GNAS1* contributes to greater fertility thus acting as a fertility enhancer.	(Nakamoto *et al*. 1998; Weinstein *et al*. 2001; Persani *et al*. 2010)
	Our model predicts that *GNAS1* will be paternally silenced in tissues affecting fertility, prediction supported by the evidence of imprinting in *GNAS1*.	
*DLX5*	Direct evidence shows that *DLX5* is imprinted in humans; maternally expressed and paternally silenced.	(Horike *et al*. 2005; Miyano *et al*. 2008)
	Mutant mice that are monoallelic for *DLX5* experience reduced fertility and early follicular exhaustion that results in premature ovarian failure. Given that expression of *DLX5* contributes to greater fertility, this locus can be classified as a fertility enhancer.	(Bouhali *et al*. 2011)
	Our model predicts that *DLX5* will be paternally silenced, prediction supported by the evidence of imprinting in *DLX5*.	
*WT1-AS*	The antisense transcript of gene *WT1* (*WT1-AS*) is imprinted in humans, in particular paternally expressed and maternally silenced. The antisense transcript antagonises the sense transcript of the same parental origin.	(Dallosso *et al*. 2004)
	*WT1* knockout mice develop without the establishment of an ovarian reserve and are non-viable. Given that expression of *WT1* contributes to establishing an ovarian reserve, this locus can be classified as a fertility enhancer and its antisense as a fertility inhibitor.	(Wilhelm & Englert 2002; Monget *et al*. 2012)
	Our model predicts that *WT1-AS* will be maternally silenced, prediction supported by the evidence of imprinting in *WT1-AS*.	

Summary of the evidence linking imprinted genes to menopause.

Moreover, we predict that those genes whose lower or greater expression reduces fertility (fertility maximisers) will exhibit random fluctuations in expression when PI but constant expression when MI. This may be the case for genes that mediate the production of oestrogen during the menstrual cycle. Hormonal deregulation results in shorter or longer menstrual cycles at the onset of peri-menopasue, ensuing the increased volatility of the cycle length (Fig. 1) (Santoro *et al*. 1996; Prior 1998, 2006; Douma *et al*. 2005). This prediction provides a first evolutionary explanation for the erratic endocrinology observed during peri-menopause. This hormonal fluctuation is responsible for many of the unpleasant symptoms experienced during peri-menopause (Avis *et al*. 2001; Gold *et al*. 2001; Prior 2006). We suggest this novel form of gene expression may also illuminate other turbulent phases of human life history in which the allocation of resources from the individual to her relatives, and *vice versa*, undergoes a major shift.

We predict that women whose ancestors evolved in populations with lower female bias in dispersal will experience greater conflict over fertility during peri-menopause (manifesting as more severe symptoms) and earlier menopause than women whose ancestors evolved in populations with higher female bias in dispersal (Fig. 2). Our model suggests that the observed variability between ethnic groups in peri-menopausal symptoms and age of menopause may be explained by ecological differences in their ancestral populations.

In particular, vaso-motor symptoms — hot flushes and night sweats — are the most common symptoms experienced by peri-menopausal women (Avis *et al*. 2001). They are caused by large fluctuations in oestrogen levels (Prior 1998). The incidence of vaso-motor symptoms is 2.5 times lower among Japanese Americans than among African Americans (after controlling for confounding factors like body mass index, smoking habits, among others) (Gold *et al*. 2000; Avis *et al*. 2001), lending support to the idea that Japanese Americans experience less severe fluctuations in oestrogen levels than do African Americans.

Premature ovarian failure refers to menopause before the age of 40 and affects 5–10% of menopausal women (Toniolo 2006). It is caused mainly by early depletion of the ovarian follicle stock due to low initial stock or accelerated atresia (Toniolo 2006; Persani *et al*. 2010). The prevalence of premature ovarian failure among Japanese Americans is ten times lower than between Hispanic and African Americans (Luborsky *et al*. 2003), lending support to the idea that Japanese Americans experience later menopause than Hispanic and African Americans. Empirical evidence does show that Japanese Americans experience later menopause (median 51.8 years) than do other ethnic groups (Gold *et al*. 2001; Henderson *et al*. 2008). The median age of menopause for Caucasian Americans, after correcting for other factors, ranges between 51.2 and 51.4 years (Bromberger *et al*. 1997; Gold *et al*. 2001); for Hispanic Americans it is 51.0 years; and for African Americans ranges between 49.3 and 51.4 (Bromberger *et al*. 1997; Henderson *et al*. 2008).

If the female ancestors of modern Japanese Americans (i.e. located in East Asia) followed their sexual partners after mating more often than the female ancestors of modern African Americans, the predictions of our model would be consistent with the evidence for: (1) Japanese Americans experiencing less severe VMS (and the underlying hormonal fluctuations) than African Americans; and (2) Japanese Americans experiencing lower incidence of premature ovarian failure and later menopause than African Americans. However, information about the ancestral ecologies of these populations is lacking. We suggest that a proper comparative analysis of patrilocality among the ancestral populations of Japanese and African Americans is a useful avenue for future research, noting that this will be complicated by the extremely diverse ancestry of African Americans. A link between ancestral ecology and age of menopause would have direct implications for the development of personalised family planning based on a woman's ethnic background.

Finally, our model suggests that genomic imprinting may play an important role in mediating the symptoms of peri-menopause and the age of menopause, and this provides opportunities for testing the model. Under our hypothesis, peri-menopausal symptoms and age at menopause would be susceptible to epigenetic modifications. In particular, we predict that experimental silencing of paternally expressed genes will alleviate peri-menopausal symptoms and delay menopause, while silencing of maternally expressed genes will hasten menopause. This is important because the timing of menopause has implications not only for a woman's fertility but also for her health in general. A delay in the onset of menopause reduces the risk of suffering osteoporosis and cardiovascular diseases, but increases the risk of suffering breast, endometrial and ovarian cancers (Hartge 2009). Therefore, overexpression of paternally expressed genes will reduce the risk of cancer while overexpression of maternally expressed genes will increase the risk of cancer. Thus, confirmation of these predictions would have direct implications for fertility treatment and indirect implications for the management of cancers and cardiovascular diseases.

For the purpose of illustration and synthesis with the existing literature on the Grandmother Hypothesis, we have focused upon female-biased dispersal as the driver of intragenomic conflict over menopause. Evidence for female-biased dispersal in ancestral human populations comes from three sources: our closest non-human relatives, contemporary hunter gatherers and genetics [reviewed by Cant & Johnstone (Cant & Johnstone 2008)]. However, some researchers favour an unbiased-dispersal interpretation [reviewed by Cant & Johnstone (2008)]. In the event that dispersal was unbiased, other sex-specific demographies could have fuelled the intragenomic conflict, by making a female more related to her neighbours via one of her parents than the other. Specifically, our qualitative results are recovered under male-biased mortality and/or variance in reproductive success, both of which are ubiquitous in humans.
