# Supplementary material for: Ecology drives intragenomic conflict over menopause
Source: Ecol Lett. 2013 Dec 9;17(2):165–74. doi: 10.1111/ele.12208 (PMC3912906; doi:10.1111/ele.12208)
Supplement: Supplementary file 1 [file ele0017-0165-sd1.pdf]

Appendix

Ecology drives intra-genomic conflict over menopause

Francisco Úbeda, Hisashi Ohtsuki & Andy Gardner

Battleground model

Age conditional consanguinities

Relatedness between a female of age t and a juvenile via the female's maternally inherited gene

The relatedness between a female of age t and a juvenile via the female's MI gene is

$$r_{Ax,\bar{M}|t} = \frac{p_{Ax,\bar{M}|t}}{p_{Mx,\bar{M}|t}} \tag{1}$$

(Úbeda and Gardner 2012) where $p_{Ax,\bar{M}|t}$ denotes the probability that the MI gene drawn from a breeding female ("aunt") of age t and a random gene (either the MI or PI copy) drawn from a neighbouring juvenile are identical by descent, and $p_{Mx,\bar{M}|t}$ denotes the probability that the MI gene drawn from a breeding female ("mother") of age t and a random gene drawn from one of her offspring are identical by descent.

The consanguinity between a female of age t and a juvenile via the female's MI gene is

$$p_{Ax,\bar{M}|t} = \tfrac{1}{2}[p_{AU,\bar{M}|t} + \alpha p_S + (1-\alpha)p_{AA,\bar{M}|t}], \tag{2}$$

and the consanguinity between a mother of age t and a juvenile via the mother's MI gene is

$$p_{Mx,\bar{M}|t} = \tfrac{1}{2}[p_{AU,\bar{M}|t} + p_S] \tag{3}$$

where p_S denotes the probability that two genes drawn at random from the same individual are identical by descent, $p_{AU\bar{M}|t}$ denotes the probability that the MI gene drawn from a breeding female of age t and a random gene (either the MI or PI copy) drawn from a neighbouring breeding male

("uncle") of any age are identical by descent, and $p_{AA\bar{M}|t}$ denotes the probability that the MI gene drawn from a female of age t and a random gene (either the MI or PI copy) drawn from a neighbouring female of any age are identical by descent. Ecologic parameters α and β correspond to the variance of reproductive success of females and males respectively.

The consanguinity between a female of age t and a male via the female's MI gene is

$$p_{AU,\bar{M}|t} = \begin{cases} \mu_m(1-d_m)(1-d_f)\frac{1}{2}[p_{AU} + \alpha p_S + (1-\alpha)p_{AA}] & \text{when } t = 1 \\ +(1-\mu_m)(1-d_f)p_{AU} & \\ & \\ \mu_m(1-d_m)\frac{1}{2}[p_{AU,\bar{M}|t} + \alpha p_S + (1-\alpha)p_{AA,\bar{M}|t}] & \text{when } t > 1 \\ +(1-\mu_m)p_{AU,\bar{M}|t} & \end{cases},$$

and the consanguinity between a female of age t and another female via the former's MI gene is

$$p_{AA,\bar{M}|t} = \begin{cases} \mu_f(1-d_f)^2\frac{1}{2}[p_{AU} + \alpha p_S + (1-\alpha)p_{AA}] & \text{when } t = 1 \\ +(1-\mu_f)(1-d_f)[\alpha p_S + (1-\alpha)p_{AA}] & \\ & \\ \mu_f(1-d_f)\frac{1}{2}[p_{AU,\bar{M}|t} + \alpha p_S + (1-\alpha)p_{AA,\bar{M}|t}] & \text{when } t > 1 \\ +(1-\mu_f)p_{AA,\bar{M}|t} & \end{cases}$$

where p_{AU} denotes the probability that a gene drawn at random from a female and a gene drawn at random from a neighbouring male are identical by descent, andp_{AA} denotes the probability that a gene drawn at random from a female and a gene drawn at random from from a neighbouring female are identical by descent. Ecologic parameters d_f and d_m correspond to dispersal in females and males while μ_f and μ_m correspond to adult mortality in females and males respectively.

Relatedness between a female of age t and a juvenile via the female's paternally inherited gene

The relatedness between a female of age t and a juvenile via her PI gene is

$$r_{Ax,\bar{P}|t} = \frac{p_{Ax,\bar{P}|t}}{p_{Mx,\bar{P}|t}} \tag{4}$$

(Úbeda and Gardner 2012) where $p_{Ax,\bar{P}|t}$ denotes the probability that the PI gene drawn from a female of age t and a random gene (either the MI or PI copy) drawn from a neighbouring juvenile

are identical by descent, and $p_{Mx,\bar{P}|t}$ denotes the probability that the PI gene drawn from a female of age t and a random gene drawn from one of her offspring are identical by descent.

The consanguinity between a female of age t and a juvenile via the female's PI gene is

$$p_{Ax,\bar{P}|t} = \tfrac{1}{2}[p_{AU,\bar{P}|t} + \alpha p_S + (1-\alpha)p_{AA,\bar{P}|t}], \tag{5}$$

and the consanguinity between a mother of age t and a juvenile via the mother's PI gene is

$$p_{Mx,\bar{P}|t} = \tfrac{1}{2}[p_{AU,\bar{P}|t} + p_S] \tag{6}$$

where $p_{AU\bar{P}|t}$ denotes the probability that the PI gene drawn from a female of age t and a random gene (either the MI or PI copy) drawn from a neighbouring male of any age are identical by descent, and $p_{AA\bar{P}|t}$ denotes the probability that the PI gene drawn from a female of age t and a random gene (either the MI or PI copy) drawn from a neighbouring female of any age are identical by descent.

The consanguinity between a female of age t and a male via the female's PI gene is

$$p_{AU,\bar{P}|t} = \begin{cases} \mu_m(1-d_m)(1-d_f)\frac{1}{2}[p_{AU} + \beta p_S + (1-\beta)p_{UU}] & \text{when } t = 1 \\ +(1-\mu_m)(1-d_f)[\beta p_S + (1-\beta)p_{UU}] & \\ & \\ \mu_m(1-d_m)\frac{1}{2}[p_{AU,\bar{P}|t} + \alpha p_S + (1-\alpha)p_{AA,\bar{P}|t}] & \text{when } t > 1 \\ +(1-\mu_m)p_{AU,\bar{P}|t} & \end{cases},$$

and the consanguinity between a female of age t and another female via the former's PI gene is

$$p_{AA,\bar{P}|t} = \begin{cases} \mu_f(1-d_f)^2\frac{1}{2}[p_{AU} + \beta p_S + (1-\beta)p_{UU}] & \text{when } t = 1 \\ +(1-\mu_f)(1-d_f)p_{AU}] & \\ & \\ \mu_f(1-d_f)\frac{1}{2}[p_{AU,\bar{P}|t} + \alpha p_S + (1-\alpha)p_{AA,\bar{P}|t}] & \text{when } t > 1 \\ +(1-\mu_f)p_{AA,\bar{P}|t} & \end{cases}$$

where p_{UU} denotes the probability that a gene drawn at random from a male and a gene drawn at random from from a neighbouring male are identical by descent.

Relatedness between a female of age t and a juvenile

We drop the parent-of-origin subscripts to represent the relatedness between a female of age t and a juvenile independent of the parent-of-origin of the female's gene. Any specific parent-of-origin

independent consanguinity can be calculated by averaging across parental origins, i.e. $p_{AU|t} = \frac{1}{2}p_{AU,\bar{M}|t} + \frac{1}{2}p_{AU,\bar{P}|t}$.

These definitions allow us to determine the relatedness between a female and a juvenile via the females's MI and PI copies for any given age of this female. Their expression is a function of p_S, p_{AA}, p_{UU}, and p_{AU} which are independent of parental origin and age. We now proceed to determine p_S, p_{AA}, p_{UU}, and p_{AU} as functions of the ecologic parameters dispersal, mating system, and adult mortality.

Age independent consanguinities

The consanguinities between two females, two males, and a female and a male are

$$\begin{aligned}
p_{AA} &= (1-\mu_f)^2 p_{AA} + (1-\mu_f)\mu_f(1-d_f)[p_{AU} + \alpha p_S + (1-\alpha)p_{AA}] \\
&\quad + \mu_f^2(1-d_f)^2\tfrac{1}{4}[2p_{AU} + (\alpha+\beta)p_S + (1-\alpha)p_{AA} + (1-\beta)p_{UU}] \\
p_{UU} &= (1-\mu_m)^2 p_{UU} + (1-\mu_m)\mu_m(1-d_m)[p_{AU} + \beta p_S + (1-\beta)p_{UU}] \\
&\quad + \mu_m^2(1-d_m)^2\tfrac{1}{4}[2p_{AU} + (\alpha+\beta)p_S + (1-\alpha)p_{AA} + (1-\beta)p_{UU}] \\
p_{AU} &= (1-\mu_f)(1-\mu_m)p_{AU} + (1-\mu_f)\mu_m(1-d_m)\tfrac{1}{2}[p_{AU} + \alpha p_S + (1-\alpha)p_{AA}] \\
&\quad + \mu_f(1-\mu_m)(1-d_f)\tfrac{1}{2}[p_{AU} + \beta p_S + (1-\beta)p_{UU}] \\
&\quad + \mu_f\mu_m(1-d_f)(1-d_m)\tfrac{1}{4}[2p_{AU} + (\alpha+\beta)p_S + (1-\alpha)p_{AA} + (1-\beta)p_{UU}] \qquad (7)
\end{aligned}$$

Finally, the consanguinity of an individual to itself is

$$p_S = \frac{1}{2}(1 + p_{AU})$$

which can be replaced in the above system of equations.

This system of equations can be solved to obtain explicit expressions for p_{AA}, p_{UU}, p_{AU} in terms of the ecologic parameters of the model. Since solutions are lengthy we do not provide them here. Substituting these solutions in the iterations provided in the previous section we can define relatedness between a female and a juvenile for a particular age of a female.

Resolution model

Directional control

As in the main text, we consider a locus that controls women's ovarian follicle stock. In particular, we consider a locus that works as a fertility inhibitor; a higher level of expression of genes at that locus reduces the number of ovarian follicle stock, thus reduces her fertility (a fertility enhancer provides an opposite case, where a higher level of expression of genes at that locus increases the number of ovarian follicle stock, hence enhances her fertility, but we will concentrate on a fertility inhibitor in the following). Let x_M and x_P be the level of expression of the MI and PI genes at that locus. We assume that the total level of expression, x, is additively determined by x_M and x_P, as $x = x_M + x_P$. In the following analysis, we assume that fertility always lies between the PI's and MI's optima, f^{*M} and f^{*P} (see Figure A2, Fertility Inhibitor), which means that the MI gene always favors a lower gene expression whereas the PI gene always favors a higher gene expression.

Suppose that each gene can choose one of the three expression levels, High (H), Normal (N), or Low (L). The number of ovarian follicles at the age peri-menopause begins is roughly 20 thousand. For simplicity, we assume that the expression at the locus considered affects the ovarian follicle stock in an additive manner; in particular, each increment in expression reduces the ovarian follicle stock by a thousand. A reduction in the ovarian follicle stock of one or two thousand results in a fertility reduction of δ_{-1} and δ_{-2} respectively. An increase in the ovarian follicle stock of one or two thousand results in a fertility increment of δ_{+1} and δ_{+2} respectively, where $-\delta_{-2} < -\delta_{-1} < 0 < \delta_{+1} < \delta_{+2}$ (see fertility matrix in Figure A1).

For the MI gene, each level of fertility enhancement/reduction $-\delta_{-2} < -\delta_{-1} < 0 < \delta_{+1} < \delta_{+2}$ corresponds to the payoff of $-\mu_{M,-2} < \mu_{M,-1} < 0 < \mu_{M,+1} < \mu_{M,+2}$, whereas for the PI gene, its payoff becomes $\mu_{P,-2} > \mu_{P,-1} > 0 > \mu_{P,+1} > \mu_{P,+2}$. Figure A1 describes the payoff matrix of the game. We allow players MI and PI to adopt a pure strategy or a mixed strategy (a strategy that cocktails the three pure strategies probabilistically). Player PI's and MI's mixed strategy is denoted by $(p_{\mathrm{H}}, p_{\mathrm{N}}, p_{\mathrm{L}})$ and $(m_{\mathrm{H}}, m_{\mathrm{N}}, m_{\mathrm{L}})$ respectively, where each element represents the probability of expression at H, N, and L levels.

Results

Because the MI's payoff monotonically increases as the total level of expression of the fertility inhibitor declines, and vice versa for the PI's payoff, the following conclusions are obvious.

The only Nash equilibrium for a fertility inhibitor is that the PI gene always plays H but the MI gene always plays L; *i.e.* $(p_{\mathrm{H}}, p_{\mathrm{N}}, p_{\mathrm{L}}) = (1, 0, 0)$ and $(m_{\mathrm{H}}, m_{\mathrm{N}}, m_{\mathrm{L}}) = (0, 0, 1)$.

We have the opposite conclusion for the opposite case, fertility enhancer (see Figure A2). The only Nash equilibrium for a fertility enhancer is that the PI gene always plays L but the MI gene always plays H; *i.e.* $(p_{\mathrm{H}}, p_{\mathrm{N}}, p_{\mathrm{L}}) = (0, 0, 1)$ and $(m_{\mathrm{H}}, m_{\mathrm{N}}, m_{\mathrm{L}}) = (1, 0, 0)$.

Stabilizing control

Menstrual cycles shorter or longer than the average during a woman's reproductive life result in a steep fertility decline (Small et al 2006). We use the term Fertility Maximizer for a locus such that fertility reduces when expression deviates from a particular value (maximum fertility). We use the term Fertility Minimizer for a locus such that fertility increases when expression deviates from a particular value (minimum fertility) (Figure A2). Henceforth, we focus on the case of a fertility maximizer but symmetric results can be obtained from considering a fertility minimizer.

The PI and MI copies can either be expressed at a high (H), normal (N), or low (L) levels as defined in the previous section. A 30-day menstrual cycle seems to be a fertility optimum (Small et al 2006) with any deviation from 30 days resulting in a fertility reduction. For simplicity, we assume that oestrogen levels during the follicular phase affect menstrual cycle length in an additive manner, in particular, each change in expression changes the menstrual cycle length by one day. A change in the menstrual cycle length of one or two days results in a fertility reduction of δ_1 and δ_2 respectively, where $\delta_2 > \delta_1 > 0$ (see fertility matrix in figure A1).

We assume that fertility always lies between the PI's and MI's optima, f^{*P} and f^{*M} (see Figure A2, Fertility Maximizer), which means that the MI gene always favors higher fertility (= a normal menstrual cycle of 30 days) whereas the PI gene favors lower fertility (= a menstrual cycle far from 30 days, either shorter or longer). We assume that each level of fertility reduction, $-\delta_2 < -\delta_1 < 0$, yields the payoff of $-\mu_{M,2} < -\mu_{M,1} < 0$ to the MI gene, whereas it yields the payoff of $\mu_{P,2} > \mu_{P,1} > 0$

to the PI gene, respectively.

Results

The proof of the following results is given in the next section.

Let $\theta_{\mathrm{P}} \equiv \mu_{P,2}/\mu_{P,1}$ and $\theta_{\mathrm{M}} \equiv \mu_{M,2}/\mu_{M,1}$.

(a)When $\theta_{\mathbf{M}} < 2$:

(a-1) When $\theta_{\mathbf{P}} < 2$: There is a unique Nash equilibrium, which is given by

$$\begin{cases} (p_{\mathrm{H}}, p_{\mathrm{N}}, p_{\mathrm{L}}) = \left(\dfrac{1}{4-\theta_{\mathrm{M}}}, \dfrac{2-\theta_{\mathrm{M}}}{4-\theta_{\mathrm{M}}}, \dfrac{1}{4-\theta_{\mathrm{M}}}\right) \\ (m_{\mathrm{H}}, m_{\mathrm{N}}, m_{\mathrm{L}}) = \left(\dfrac{1}{4-\theta_{\mathrm{P}}}, \dfrac{2-\theta_{\mathrm{P}}}{4-\theta_{\mathrm{P}}}, \dfrac{1}{4-\theta_{\mathrm{P}}}\right). \end{cases} \tag{8}$$

(a-2) When $\theta_{\mathbf{P}} = 2$: There is a continuum of Nash equilibria given by

$$\begin{cases} (p_{\mathrm{H}}, p_{\mathrm{N}}, p_{\mathrm{L}}) = (p, 1-2p, p) \qquad \left(\dfrac{1}{4-\theta_{\mathrm{M}}} \le p \le \dfrac{1}{2}\right) \\ (m_{\mathrm{H}}, m_{\mathrm{N}}, m_{\mathrm{L}}) = (0.5, 0, 0.5)\,. \end{cases} \tag{9}$$

(a-3) When $\theta_{\mathbf{P}} > 2$: There is a unique Nash equilibria given by

$$\begin{cases} (p_{\mathrm{H}}, p_{\mathrm{N}}, p_{\mathrm{L}}) = (0.5, 0, 0.5) \\ (m_{\mathrm{H}}, m_{\mathrm{N}}, m_{\mathrm{L}}) = (0.5, 0, 0.5)\,. \end{cases} \tag{10}$$

(b)When $\theta_{\mathbf{M}} = 2$: There is a continuum of Nash equilibria given by

$$\begin{cases} (p_{\mathrm{H}}, p_{\mathrm{N}}, p_{\mathrm{L}}) = (0.5, 0, 0.5) \\ (m_{\mathrm{H}}, m_{\mathrm{N}}, m_{\mathrm{L}}) = (m, 1-2m, m) \qquad \left(0 \le m \le \min\left[\dfrac{1}{4-\theta_{\mathrm{P}}}, \dfrac{1}{2}\right]\right). \end{cases} \tag{11}$$

(c)When $\theta_{\mathbf{M}} > 2$: There is a continuum of Nash equilibria given by

$$\begin{cases} (p_{\mathrm{H}}, p_{\mathrm{N}}, p_{\mathrm{L}}) = (p, 0, 1-p) \qquad \left(\dfrac{1}{\theta_{\mathrm{M}}} \le p \le 1-\dfrac{1}{\theta_{\mathrm{M}}}\right) \\ (m_{\mathrm{H}}, m_{\mathrm{N}}, m_{\mathrm{L}}) = (0, 1, 0)\,. \end{cases} \tag{12}$$

Overall, if the effect of 2-day deviation from the normal menstrual cycle (=30 days) is more than twice as detrimental as 1-day deviation to the MI gene (*i.e.* $\theta_{\mathrm{M}} = \mu_{M,2}/\mu_{M,1} > 2$), we predict that the expression level of the MI gene is stable but that of the PI gene is very much randomized, which qualitatively agrees with the result in the main text.

Proofs of analytical results

The payoff of the PI gene is given as

$$u_P = \underbrace{(m_{\mathrm{N}} + \theta_{\mathrm{P}} m_{\mathrm{L}})}_{\text{(I)}} p_{\mathrm{H}} + \underbrace{(m_{\mathrm{H}} + m_{\mathrm{L}})}_{\text{(II)}} p_{\mathrm{N}} + \underbrace{(m_{\mathrm{N}} + \theta_{\mathrm{P}} m_{\mathrm{H}})}_{\text{(III)}} p_{\mathrm{L}}, \tag{13}$$

where u_P is already normalized by the factor, $\mu_{P,1}$. Similarly, the payoff of the MI gene is given as

$$u_M = -\underbrace{(p_{\mathrm{N}} + \theta_{\mathrm{M}} p_{\mathrm{L}})}_{\text{(i)}} m_{\mathrm{H}} - \underbrace{(p_{\mathrm{H}} + p_{\mathrm{L}})}_{\text{(ii)}} m_{\mathrm{N}} - \underbrace{(p_{\mathrm{N}} + \theta_{\mathrm{M}} p_{\mathrm{H}})}_{\text{(iii)}} m_{\mathrm{L}}. \tag{14}$$

where u_M is already normalized by the factor, $\mu_{M,1}$.

For a combination of mixed strategies, $(p_{\mathrm{H}}, p_{\mathrm{N}}, p_{\mathrm{L}})$ and $(m_{\mathrm{H}}, m_{\mathrm{N}}, m_{\mathrm{L}})$, to be a Nash equilibrium of the game considered, each must be a best response to the other. In other words, they must satisfy the following relation:

$$(p_{\mathrm{H}}, p_{\mathrm{N}}, p_{\mathrm{L}}) = \begin{cases} (1,0,0) & \text{if (I)>(II),(III)} \\ (0,1,0) & \text{if (II)>(I),(III)} \\ (0,0,1) & \text{if (III)>(I),(II)} \\ (*,*,0) & \text{if (I)=(II)>(III)} \\ (*,0,*) & \text{if (I)=(III)>(II)} \\ (0,*,*) & \text{if (II)=(III)>(I)} \\ (*,*,*) & \text{if (I)=(II)=(III)} \end{cases} \tag{15a}$$

$$(m_{\mathrm{H}}, m_{\mathrm{N}}, m_{\mathrm{L}}) = \begin{cases} (1,0,0) & \text{if (i)<(ii),(iii)} \\ (0,1,0) & \text{if (ii)<(i),(iii)} \\ (0,0,1) & \text{if (iii)<(i),(ii)} \\ (*,*,0) & \text{if (i)=(ii)<(iii)} \\ (*,0,*) & \text{if (i)=(iii)<(ii)} \\ (0,*,*) & \text{if (ii)=(iii)<(i)} \\ (*,*,*) & \text{if (i)=(ii)=(iii)} \end{cases} \tag{15b}$$

Here $*$ represents a wild-card.

First, suppose (I)>(II),(III) holds. Then $(p_{\mathrm{H}}, p_{\mathrm{N}}, p_{\mathrm{L}}) = (1,0,0)$ must hold, leading to $((\mathrm{i}), (\mathrm{ii}), (\mathrm{iii})) = (0, 1, \theta_{\mathrm{M}})$, which leads to $(m_{\mathrm{H}}, m_{\mathrm{N}}, m_{\mathrm{L}}) = (1,0,0)$, leading to $((\mathrm{I}), (\mathrm{II}), (\mathrm{III})) = (0, 1, \theta_{\mathrm{P}})$. Contradiction.

Second, suppose (II)>(I),(III) holds. Then $(p_{\rm H}, p_{\rm N}, p_{\rm L}) = (0, 1, 0)$ must hold, leading to $((\text{i}), (\text{ii}), (\text{iii})) = (1, 0, 1)$, which leads to $(m_{\rm H}, m_{\rm N}, m_{\rm L}) = (0, 1, 0)$, leading to $((\text{I}), (\text{II}), (\text{III})) = (1, 0, 1)$. Contradiction.

Third, suppose (III)>(I),(II) holds. Then $(p_{\rm H}, p_{\rm N}, p_{\rm L}) = (0, 0, 1)$ must hold, leading to $((\text{i}), (\text{ii}), (\text{iii})) = (\theta_{\rm M}, 1, 0)$, which leads to $(m_{\rm H}, m_{\rm N}, m_{\rm L}) = (0, 0, 1)$, leading to $((\text{I}), (\text{II}), (\text{III})) = (\theta_{\rm P}, 1, 0)$. Contradiction.

Fourth, suppose (I)=(II)>(III) holds. Then $(p_{\rm H}, p_{\rm N}, p_{\rm L}) = (*, *, 0)$ must hold, leading to $((\text{i}), (\text{ii}), (\text{iii})) = (p_{\rm N}, p_{\rm H}, p_{\rm N} + \theta_{\rm M} p_{\rm H})$.

- If $p_{\rm N} < p_{\rm H}$ holds, we have (i)<(ii),(iii), thus $(m_{\rm H}, m_{\rm N}, m_{\rm L}) = (1, 0, 0)$, leading to $((\text{I}), (\text{II}), (\text{III})) = (0, 1, \theta_{\rm P})$. Contradiction.

- If $p_{\rm N} = p_{\rm H}$ holds, we have (i)=(ii)<(iii), thus $(m_{\rm H}, m_{\rm N}, m_{\rm L}) = (*, *, 0)$, leading to $((\text{I}), (\text{II}), (\text{III})) = (m_{\rm N}, m_{\rm H}, m_{\rm N} + \theta_{\rm P} m_{\rm H})$, but from the original assumption we must have $m_{\rm N} = m_{\rm H} > m_{\rm N} + \theta_{\rm P} m_{\rm H}$, which can never be the case.

- If $p_{\rm N} > p_{\rm H}$ holds, we have (ii)<(i),(iii), thus $(m_{\rm H}, m_{\rm N}, m_{\rm L}) = (0, 1, 0)$, leading to $((\text{I}), (\text{II}), (\text{III})) = (1, 0, 1)$. Contradiction.

Fifth, suppose (I)=(III)>(II) holds. Then $(p_{\rm H}, p_{\rm N}, p_{\rm L}) = (*, 0, *)$ must hold, leading to $((\text{i}), (\text{ii}), (\text{iii})) = (\theta_{\rm M} p_{\rm L}, 1, \theta_{\rm M} p_{\rm H})$.

<u>For $\theta_{\rm M} < 2$:</u>

- If $p_{\rm L} < p_{\rm H}$ holds, we have (i)<(ii),(iii), thus $(m_{\rm H}, m_{\rm N}, m_{\rm L}) = (1, 0, 0)$, leading to $((\text{I}), (\text{II}), (\text{III})) = (0, 1, \theta_{\rm P})$. Contradiction.

- If $p_{\rm L} = p_{\rm H}$ holds, we have (i)=(iii)<(ii), thus $(m_{\rm H}, m_{\rm N}, m_{\rm L}) = (*, 0, *)$, leading to $((\text{I}), (\text{II}), (\text{III})) = (\theta_{\rm P} m_{\rm L}, 1, \theta_{\rm P} m_{\rm H})$. From the original assumption we must have $\theta_{\rm P} m_{\rm L} = \theta_{\rm P} m_{\rm H} > 1$, which has the solution $(m_{\rm H}, m_{\rm N}, m_{\rm L}) = (1/2, 0, 1/2)$ if and only if $\theta_{\rm P} > 2$.

- If $p_{\rm L} > p_{\rm H}$ holds, we have (iii)<(i),(ii), thus $(m_{\rm H}, m_{\rm N}, m_{\rm L}) = (0, 0, 1)$, leading to $((\text{I}), (\text{II}), (\text{III})) = (\theta_{\rm P}, 1, 0)$. Contradiction.

<u>For $\theta_{\rm M} = 2$:</u>

- If $p_L < p_H$ holds, we have (i)<(ii),(iii), thus $(m_H, m_N, m_L) = (1, 0, 0)$, leading to $((I), (II), (III)) = (0, 1, \theta_P)$. Contradiction.

- If $p_L = p_H$ holds (hence $(p_H, p_N, p_L) = (0.5, 0, 0.5)$), we have (i)=(ii)=(iii), thus $(m_H, m_N, m_L) = (*, *, *)$, suggesting no restrictions on m's, but from the original assumption we must have $m_N + \theta_P m_L = m_N + \theta_P m_H > m_H + m_L$, leading to $(m_H, m_N, m_L) = (m, 1 - 2m, m)$ $(0 \leq m \leq 1/2$ and $m < 1/(4 - \theta_P))$.

- If $p_L > p_H$ holds, we have (iii)<(i),(ii), thus $(m_H, m_N, m_L) = (0, 0, 1)$, leading to $((I), (II), (III)) = (\theta_P, 1, 0)$. Contradiction.

<u>For $\theta_M > 2$:</u>

- If $p_L < 1/\theta_M$ holds, we have (i)<(ii),(iii), thus $(m_H, m_N, m_L) = (1, 0, 0)$, leading to $((I), (II), (III)) = (0, 1, \theta_P)$. Contradiction.

- If $p_L = 1/\theta_M$ holds (hence $(p_H, p_N, p_L) = (1 - 1/\theta_M, 0, 1/\theta_M)$), we have (i)=(ii)<(iii), thus $(m_H, m_N, m_L) = (*, *, 0)$, leading to $((I), (II), (III)) = (m_N, m_H, m_N + \theta_P m_H)$, but from the original assumption we must have $m_N = m_N + \theta_P m_H > m_H$, leading to $(m_H, m_N, m_L) = (0, 1, 0)$.

- If $1/\theta_M < p_L < 1 - 1/\theta_M$ holds, we have (ii)<(i),(iii), thus $(m_H, m_N, m_L) = (0, 1, 0)$, leading to $((I), (II), (III)) = (1, 0, 1)$, which agrees with the original assumption.

- If $p_L = 1 - 1/\theta_M$ holds (hence $(p_H, p_N, p_L) = (1/\theta_M, 0, 1 - 1/\theta_M)$), we have (ii)=(iii)<(i), thus $(m_H, m_N, m_L) = (0, *, *)$, leading to $((I), (II), (III)) = (m_N + \theta_P m_L, m_L, m_N)$, but from the original assumption we must have $m_N + \theta_P m_L = m_N > m_L$, leading to $(m_H, m_N, m_L) = (0, 1, 0)$.

- If $p_L > 1 - 1/\theta_M$ holds, we have (iii)<(i),(ii), thus $(m_H, m_N, m_L) = (0, 0, 1)$, leading to $((I), (II), (III)) = (\theta_P, 1, 0)$. Contradiction.

Sixth, suppose (II)=(III)>(I) holds. Then $(p_H, p_N, p_L) = (0, *, *)$ must hold, leading to $((i), (ii), (iii)) = (p_N + \theta_M p_L, p_L, p_N)$.

- If $p_N < p_L$ holds, we have (iii)<(i),(ii), thus $(m_H, m_N, m_L) = (0, 0, 1)$, leading to $((I), (II), (III)) = (\theta_P, 1, 0)$. Contradiction.

- If $p_{\mathrm{N}} = p_{\mathrm{L}}$ holds, we have (ii)=(iii)<(i), thus $(m_{\mathrm{H}}, m_{\mathrm{N}}, m_{\mathrm{L}}) = (0, *, *)$, leading to $((\mathrm{I}), (\mathrm{II}), (\mathrm{III})) = (m_{\mathrm{N}} + \theta_{\mathrm{P}} m_{\mathrm{L}}, m_{\mathrm{L}}, m_{\mathrm{N}})$, but from the original assumption we must have $m_{\mathrm{N}} = m_{\mathrm{L}} > m_{\mathrm{N}} + \theta_{\mathrm{P}} m_{\mathrm{L}}$, which can never be the case.

- If $p_{\mathrm{N}} > p_{\mathrm{L}}$ holds, we have (ii)<(i),(iii), thus $(m_{\mathrm{H}}, m_{\mathrm{N}}, m_{\mathrm{L}}) = (0, 1, 0)$, leading to $((\mathrm{I}), (\mathrm{II}), (\mathrm{III})) = (1, 0, 1)$. Contradiction.

Seventh, suppose (I)=(II)=(III) holds.

<u>For $\theta_{\mathrm{P}} < 2$:</u> We have $(m_{\mathrm{H}}, m_{\mathrm{N}}, m_{\mathrm{L}}) = \frac{1}{4-\theta_{\mathrm{P}}}(1, 2 - \theta_{\mathrm{P}}, 1)$. As each probability is strictly positive, from eq.(15b) we must have (i)=(ii)=(iii), leading to $(p_{\mathrm{H}}, p_{\mathrm{N}}, p_{\mathrm{L}}) = \frac{1}{4-\theta_{\mathrm{M}}}(1, 2 - \theta_{\mathrm{M}}, 1)$.

<u>For $\theta_{\mathrm{P}} = 2$:</u> We have $(m_{\mathrm{H}}, m_{\mathrm{N}}, m_{\mathrm{L}}) = (0.5, 0, 0.5)$. From eq.(15b) we must have (i)=(iii)≤(ii), leading to $p_{\mathrm{N}} + \theta_{\mathrm{M}} p_{\mathrm{L}} = p_{\mathrm{N}} + \theta_{\mathrm{M}} p_{\mathrm{H}} \leq p_{\mathrm{H}} + p_{\mathrm{L}}$, which is equivalent to $(p_{\mathrm{H}}, p_{\mathrm{N}}, p_{\mathrm{L}}) = (p, 1 - 2p, p)$ $(1/(4 - \theta_{\mathrm{M}}) \leq p \leq 1/2)$.

<u>For $\theta_{\mathrm{P}} > 2$:</u>

There is no m that satisfies the assumption, (I)=(II)=(III).

Figure A1.- Fertility and payoff matrices. The first column corresponds to the game played by a fertility inhibitor that determines a woman's oocyte stock. Each matrix has three entries corresponding to the levels of expression lower (L), normal (N), and higher (H). The cells in the fertility matrix indicate the change in fertility driven by the change in the oocyte stock at the beginning of peri-menopause (background figures represent number of oocytes in thousands). The cells in the payoff matrix indicate the change in payoff for the MI (shaded region) and the PI (non-shaded region). The second column corresponds to the game played by a fertility disruptor that determines the length of a woman's menstrual cycle. The cells in the fertility matrix indicate the change in fertility driven by the change in menstrual cycle length (background figures represent number of days). The cells in the payoff matrix indicate the change in payoff for the MI (shaded region) and the PI (non-shaded region).

Figure A2.- Payoff as a function of expression and fertility. Payoff as a function of fertility V(f), fertility as a function of gene expression F(z), and payoff as a function of gene expression U(z). We start by considering when gene expression x exerts a directional control of fertility A. (A.1) Fertility inhibitor when the relation between fertility and gene expression is negative. (A.2) Fertility enhancer when the relation between fertility and gene expression is positive. Then we consider when gene expression y exerts a stabilizing/destabilizing control of fertility B. (B.1) Fertility maximizer when fertility reaches a maximum. (B.2) Fertility minimizer when fertility reaches a minimum. Asterisks represent maximum payoff.
